# Dietary Phospholipid-Bound Conjugated Linoleic Acid and Docosahexaenoic Acid Incorporation Into Fetal Liver and Brain Modulates Fatty Acid and *N*-Acylethanolamine Profiles

**DOI:** 10.3389/fnut.2022.834066

**Published:** 2022-03-10

**Authors:** Elisabetta Murru, Gianfranca Carta, Claudia Manca, Asgeir Saebo, Michele Santoni, Rafaela Mostallino, Marco Pistis, Sebastiano Banni

**Affiliations:** ^1^Department of Biomedical Sciences, University of Cagliari, Cagliari, Italy; ^2^Innolipid AS, Ålesund, Norway; ^3^Neuroscience Institute, Section of Cagliari, National Research Council of Italy (CNR), Cagliari, Italy; ^4^Unit of Clinical Pharmacology, University Hospital, Cagliari, Italy

**Keywords:** conjugated linoleic acid (CLA), docosahexaenoic acid (DHA), *N*-acylethanolamines (NAEs), fetal brain, maternal nutrition

## Abstract

We evaluated whether maternal intake of conjugated linoleic acid (CLA) and docosahexaenoic acid (DHA) in the phospholipid (PL) form (CLA-DHA PL) affects maternal and fetal brain and liver fatty acids (FAs) profile and the biosynthesis of FA-derived bioactive lipid mediators *N*-acylethanolamines (NAEs) involved in several neurophysiological functions. We fed rat dams during the first 2/3 of their pregnancy a CLA-DHA PL diet containing PL-bound 0.5% CLA and 0.2% DHA. FA and NAE profiles were analyzed in maternal and fetal liver and brain by Liquid Chromatography diode array detector (LC-DAD) and MS/MS in line. We found that CLA and DHA crossed the placenta and were readily incorporated into the fetal liver and brain. CLA metabolites were also found abundantly in fetal tissues. Changes in the FA profile induced by the CLA-DHA PL diet influenced the biosynthesis of NAE derived from arachidonic acid (ARA; *N*-arachidonoylethanolamine, AEA) and from DHA (*N*-docosahexaenoylethanolamine, DHEA). The latter has been previously shown to promote synaptogenesis and neuritogenesis. The reduced tissue n6/n3 ratio was associated to a significant decrease of AEA levels in the fetal and maternal liver and an increase of DHEA in the fetal and maternal liver and in the fetal brain. Maternal dietary CLA-DHA PL by promptly modifying fetal brain FA metabolism, and thereby, increasing DHEA, might represent an effective nutritional strategy to promote neurite growth and synaptogenesis and protect the offspring from neurological and psychiatric disorders with neuroinflammatory and neurodegenerative basis during the critical prenatal period.

## Introduction

The fat composition in the fetus is of major importance, as the intrauterine requirement of n6 and n3 polyunsaturated fatty acids (PUFAs) in the human fetus development during the last trimester and the early weeks of life is 400 and 50 mg/kg/day, respectively (1). In fact, cerebral tissue, where lipids make up to 50% of the dry weight, has around half the total lipid content composed of long-chain PUFA (LC-PUFA), of which arachidonic acid (ARA, 20:4n6) and docosahexaenoic acid (DHA, 22:6n3) are metabolically the most important for the development of the central nervous system (CNS) the body growth and the synthesis of bioactive molecules (2).

The placenta and the fetus, owing to the low activities of the delta 5- and delta 6-desaturase enzymes, cannot synthesize LC-PUFA from the essential fatty acids (FAs) (3); consequently, the considerable requirements of these LC-PUFA in the fetus must be provided by their placental transfer through lipoprotein receptors (4–6) and lipase activities (7–9), which facilitate the release of FAs and their subsequent transport to the fetal liver. A placental selectivity and high affinity for the transport of individual FA to the fetus has been reported (10–12), explaining why the concentrations of some LC-PUFA, as ARA and DHA, are greater in the fetal than maternal circulation (10, 13–15). Haggarty et al. (16) found a selective preferential placental transport of DHA with order of preference DHA > ARA > alpha linolenic acid (ALA, 18:3n3) > linoleic acid (LA, 18:2n6) (16, 17).

Several studies showed that maternal intake of LC-PUFAn3, in particular, the DHA, during pregnancy plays a crucial role in the CNS development leading to positive effects on visual, mental, and psychomotor development (18–23). It was shown that incorrect nutritional management of the mother during fetal brain development might lower the threshold for neurological disorders later in life (24, 25).

Different approaches have been suggested to optimize the maternal intake of some FAs to increase their availability to the fetus, creating a balance among FAs important to prevent undesirable consequences to the newborns (26).

An unusual FA, the conjugated linoleic acid (CLA), which has several nutritional properties at the peripheral level (27–31), has been demonstrated to cross the hematoencephalic barrier (BBB) in both experimental animals (32) and humans (33) with a promising positive impact on neurological disorders (34), such as adrenoleukodystrophy (33, 35). CLA is a positional and geometric isomer of LA which is present in meat and dairy products of ruminants and synthesized endogenously in non-ruminants and humans (36–38). We have previously demonstrated that CLA is incorporated and metabolized into brain tissue (32). The most of the studies that investigated CLA described its capacity to improve protection against neuroinflammation (39, 40), induce a re-balance of the dopaminergic neuronal function (41), and enhance the synaptogenesis and neuritogenesis (41–43). This reinforces the idea that CLA can be a very good alimentary support in the prevention of psychiatric disorders with neuroinflammatory and neurodegenerative basis (34, 41). In fact, chronic dietary CLA intake can reduce prostaglandin E2 in the peripheral tissues and CNS (40). In addition, it has been shown that CLA protects mouse cortical neurons from glutamate excitotoxicity (39).

Surprisingly, despite the significant number of studies that investigated the positive biological effects of CLA in the brain, there is only a limited amount of data on the importance of CLA supplementation during the gestational period to improve the cerebral functions in offspring. Recently, Queiroz et al. (44) investigated the impact of different CLA concentrations (1 and 3%) mixed into the maternal diet during gestation and lactation in an experimental model. The authors analyzed the reflex responses and cognitive functions of the offspring from the 1st to the 21st day after birth, and the FA profiles in the breast milk and in the offspring's brain were also quantified. The milk with 3% dietary CLA presented an increase in PUFA and a decrease in monounsaturated fatty acid (MUFA), and the amount of CLA was greater in the two CLA groups than the control group, while maternal and both offspring's brains presented only moderated CLA levels. The maternal CLA diet induced anticipated reflex maturation and improved learning and memory in the offspring. However, Queiroz did not analyze the CLA incorporation in fetal tissues, which we suppose is crucial to obtain beneficial effects in the offspring (44). Reynolds et al. observed that supplementation during pregnancy and lactation of CLA c9, t11 reverted reproductive and metabolic dysfunction of the offspring induced by a maternal high fat diet (45).

This study aimed to investigate the bioavailability and incorporation of CLA in the liver and brain of the fetus of Sprague Dawley rat dams fed, during the gestational period, with a diet supplemented with CLA (0.5% of the diet) and DHA (0.2% of the diet) in the form of phospholipid (PL). We supposed that CLA and DHA esterified to PL (CLA-DHA PL) could further increase their bioavailability into PL of the brain. Accordingly, we previously showed that the dietary intake of FA in the PL form, where DHA, was around 0.2% of the diet, is more effective in increasing its concentrations in the brain than the triglyceride form (46, 47). On the other hand, the percentage of dietary CLA (0.5%) was chosen as the lowest amount able to exert its biological activities, as demonstrated in different experimental models (31). Overall, these dietary concentrations may correspond to dietary intake in humans of 3g/d for CLA (48) and 800 mg/d of DHA (47), both levels that may easily be reached by dietary supplementation.

Both FAs have been shown to exert their biological activities by influencing FA metabolism and consequent changes of a series of bioactive lipid mediators, namely, endocannabinoid-related molecules (47, 49, 50). Therefore, besides the analysis of liver and brain FA profiles, we also analyzed in fetal and maternal liver and brain the influence of dietary CLA-DHA PL formulation on the biosynthesis of bioactive metabolites, such as the endocannabinoid *N-*arachidonoylethanolamine or anandamide (AEA) and the congeners *N-*acylethanolamines (NAEs). Different studies have demonstrated that CLA influences the synthesis of these molecules and is an avid ligand of Peroxisome proliferator-activated receptor (PPAR) alpha whose activation is important for the pathophysiologic response to neuroinflammatory events (34, 50, 51).

## Materials and Methods

### Experimental Diets

The diets were manufactured at Charles River Laboratories Italia Srl. The control diet (CRTL) was based on the AIN-93G formulation containing 7% of total fat as soybean oil, which provides 14% of the total energy (%en), whereas the experimental diet contains 6% soybean oil + 1% mixture CLA-DHA (CLA 0.5% and DHA 0.2% of the diet; CLA-DHA PL). These two diets were equilibrated in % of FA, with the only variation represented by CLA and DHA levels provided as an ester of PLs, precisely phosphatidylcholine (PC). This experimental formulation was provided by INNOLIPID AS, Ålesund, Norway.

Table 1 reports the FA composition of both diets, expressed in % of the diet.

**Table 1:** Composition of control (CTRL) and experimental [conjugated linoleic acid-docosahexaenoic acid in phospholipids (CLA-DHA PL)] diets administered to rat dams from day zero to 16 of the gestational period.

**% of the diets**	**CRTL**	**CLA-DHA PL**
ALA, 18:3n3	0.2	0.2
EPA, 20:5n3	0.0	0.1
DHA, 22:6n3	0.0	0.2
LA, 18:2n6	1.6	1.3
OA, 18:1n9	2.1	1.9
CLA, CD18:2	0.0	0.5
PA, 16:0	0.4	0.6
SA, 18:0	0.1	0.2
PUFAn3	0.2	0.4
PUFAn6	1.6	1.3
PUFAn6/n3	7.6	3.1

*The Table reports the main fatty acids (FAs) present in the diets, which are expressed in % of the diet*.

### Animals and Sample Collection

Experiments were approved by the Animal Ethics Committees of the University of Cagliari and were carried out in accordance with the European Directive on the protection of animals used for scientific purposes (2010/63/EU).

Sprague Dawley rats were housed in groups in standard conditions of temperature (21 ± 1°C) and humidity (60%) under a 12 h light/dark cycle (lights on at 7:00 a.m.) with food and water available *ad libitum*. Eight female rats were mated at the age of 3 months with an initial weight of 230–250 g. The first day after the copulation, which was confirmed through the presence of the vaginal plug, was defined as gestational day zero. Dams were randomly assigned to two experimental groups: the first group received a control (CRTL) diet, whereas the second group received a diet enriched with CLA and DHA in the form of PL (CLA-DHA PL) *ad libitum* from day zero to 16 of the gestational period. We did not observe any significant difference in the food intake and body weight between the two groups (data not shown).

Before sacrifice, the dams were fasted for 12 h and were subsequently euthanized. Liver, plasma, and different cerebral areas, such as the hypothalamus and hippocampus, were then collected and stored at −80°C for lipid analyses. Four fetuses were also collected from each mother, and their livers and brains *in toto* were pooled for lipid analyses.

### Lipid Analyses

#### FAs Analysis

Total lipids were extracted from fetal and maternal liver and brain samples according to the method of Folch et al. (52). Total lipid quantification was performed by the method of Chiang et al. (53). Aliquots of the lipid fraction were mildly saponified using a procedure in order to obtain unsaturated FAs for High Performance Liquid Chromatography (HPLC) analysis (54). The reagents were HPLC grade and purchased from Sigma Chemicals Co. (St. Louis, MO, USA). The separation of FAs was carried out using an Agilent 1100 HPLC System (Agilent, Palo Alto, CA, USA) equipped with a diode array detector (DAD). A C18 Inertsil 5 ODS-2 Chrompack Column (Chrompack International BV, Middleburg, The Netherlands) with 5 μm particle size and 150 × 4.6 mm was used with a mobile phase of CH_3_CN/H_2_O/CH_3_COOH (70/30/0.12, v/v/v) at a flow rate of 1.5 ml/min (55). Saturated FAs (SFAs) were measured as fatty acid methyl esters (FAMEs) by a gas chromatograph (Agilent, Model 6890, Palo Alto) equipped with a flame ionization detector (FID); a 100 m HP-88 fused capillary column (Agilent, Palo Alto) was used. Data were acquired by the Agilent ChemStation software system (49).

#### NAEs Analysis

Aliquots of the lipid fraction were used for the quantification of NAE compounds. Deuterated NAE and congeners were added as internal standards to the samples before extraction for quantification by isotope dilution. Internal deuterated standards [2H]_8_AEA, [2H]_2_OEA, [2H]_4_PEA, [2H]_3_SEA were purchased from Cayman Chemicals (MI, USA). NAE quantification was carried out by an Agilent 1100 HPLC system (Agilent, Palo Alto) equipped with a mass spectrometry (MS) Agilent Technologies QQQ triple quadrupole 6420 with electrospray ionization (ESI) source, using positive mode (ESI+). A C18 Zorbax Eclipse Plus Column (Agilent, Palo Alto) with 5 μm particle size and 50 × 4.6 mm was used with a mobile phase of CH_3_OH/H_2_O/CHOOH (80/20/0.1, v/v/v) at a flow rate of 0.5 ml/min (49).

Data were acquired by the MassHunter Workstation acquisition software (version B.08.02), analyzed with MassHunter software for qualitative (version B.08.00 SP1) and quantitative analyses (version B.09.00). NAE compounds were expressed as pmol/g tissues.

### Statistical Analysis

Quantitative data are presented as mean ± SEM. Statistical differences between experimental and control groups were evaluated by Student's *t*-test and statistical significances were indicated as follows: ^****^p ≤ 0.0001; ^***^p ≤ 0.001; ^**^p ≤ 0.01; ^*^p ≤ 0.05. Correlation studies between circulating levels of maternal CLA and its CD18:3 and CD16:2 metabolites with the respective levels in fetal liver were done using the Spearman correlation coefficient with a 95% CI. Data were analyzed using GraphPad Prism 6.0 (GraphPad Software Inc., La Jolla, CA, USA).

## Results

### Accumulation of CLA, Its Metabolites, and DHA in Liver and Brain Tissue of Rat Dams and Their Fetuses

**Figure 1:**
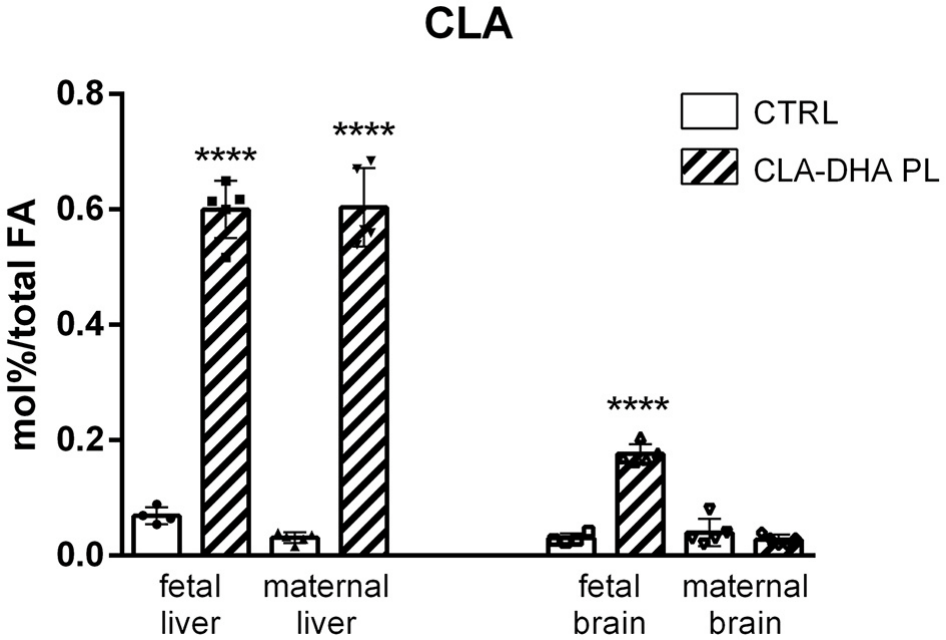
Incorporation of conjugated linoleic acid (CLA), expressed in mol%/total FA, in liver and brain of rat dams fed with control (CRTL) and experimental (CLA-DHA PL) diets from day 0 to 16 of the gestational period, and their fetuses (*n* = 4/group). Statistical significance is depicted as follows: ****p ≤ 0.0001.

Figure 1 represents the different incorporation of CLA in the liver and brain of dams and their fetuses after 16 days of dietary intake with CRTL and CLA-DHA PL diets. The values are indicated as mol% of CLA with respect to total FA. The data showed a significantly higher CLA incorporation in the liver of rats supplemented with the CLA/DHA-PL diet with respect to the CRTL group; particularly, CLA incorporation was similar in rat dams and their fetus. Unexpectedly, fetal brain displayed increased levels of CLA in CLA-DHA PL, significantly higher than CTRL groups, while in the maternal brain, we did not detect any significant CLA incorporation, confirming that FA incorporation in the adult rat brain is very difficult (56, 57). In rat dams, we limited our analysis to two brain areas, the hypothalamus and hippocampus. Both regions displayed similar variations between the treatments in all the analyses performed; therefore, we here report only data on the hypothalamus. In the fetuses, we report data on the whole brain, as it was not feasible to isolate different brain areas.

**Figure 2:**
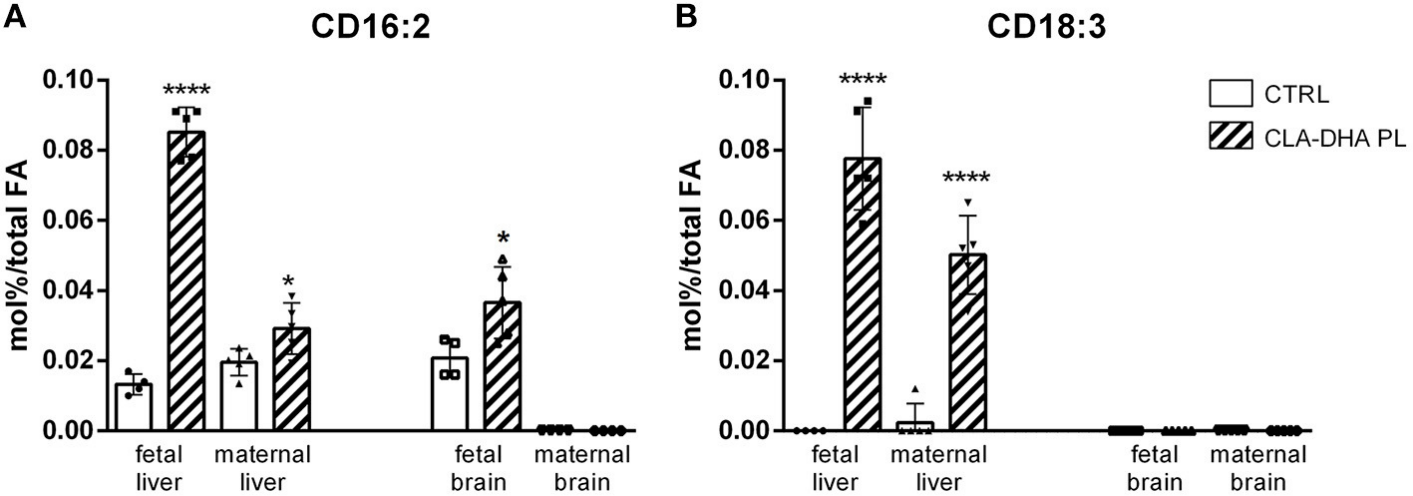
Incorporation of CD16:2 **(A)** and CD18:3 **(B)**, expressed in mol%/total FA, in liver and brain of rat dams fed with control (CRTL) and experimental (CLA-DHA PL) diets from day 0 to 16 of the gestational period, and their fetuses (*n* = 4/group). Statistical significance is depicted as follows: ****p ≤ 0.0001 and *p ≤ 0.05.

Two conjugated diene (CD) metabolites of CLA were detected, the CD 16:2 and CD 18:3, produced from peroxisomal beta-oxidation and from CLA desaturation by delta-6 desaturase activity, respectively. The CLA-DHA PL diet increased the CD16:2 levels in the liver and brain of fetuses (Figure 2A) and in mothers only in the liver. CD18:3 was increased in maternal liver with respect to control group and in the fetal liver was detected only after CLA-DHA PL diet. In fetal and maternal brains CD18:3 was not detected, as visualized in Figure 2B. As expected, similarly to what showed for CLA, these metabolites were not detected in the hypothalamus and hippocampus of dams.

**Figure 3:**
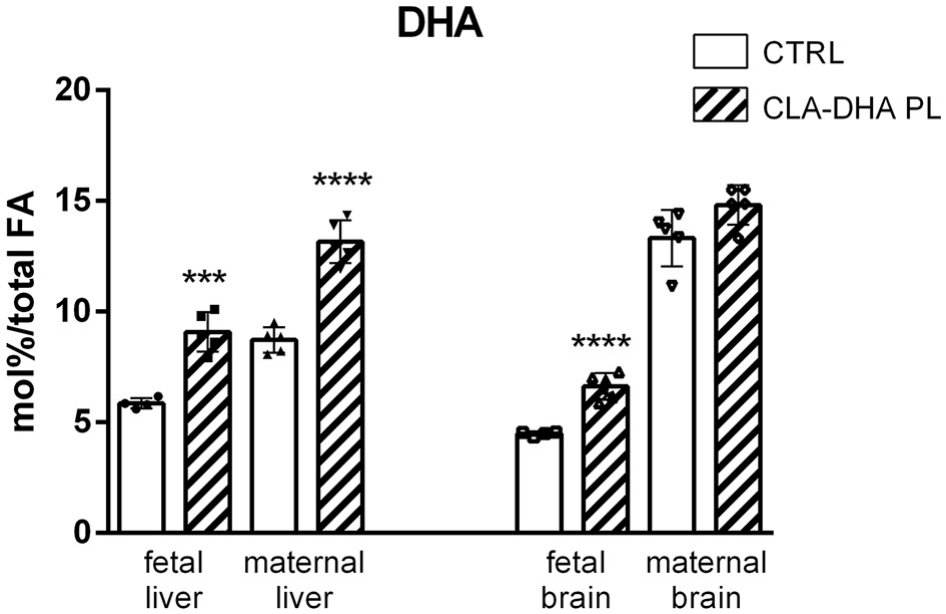
Incorporation of DHA, expressed in mol%/total FA, in liver and brain of rat dams fed with control (CRTL) and experimental (CLA-DHA PL) diets from day 0 to 16 of the gestational period, and their fetuses (*n* = 4/group). Statistical significance is depicted as follows: ****p ≤ 0.0001 and ***p ≤ 0.001.

Figure 3 displays the different incorporation of DHA in the liver and brain of dams and their fetuses. As for CLA, there was a significant increase of DHA incorporation in fetal liver and brain of the rats supplemented with the CLA-DHA PL diet when compared to the CRTL groups. Brain DHA levels did not change significantly in dams, while we detected a significant increase in the liver.

Table 2 reports the FA profile, expressed in mol%/total FA, in liver and brain of rat dams fed with CRTL or CLA-DHA PL diets and their fetuses. Dietary CLA-DHA PL determined an increment of PUFAn3 and a simultaneous reduction of PUFAn6 compared to the CRTL diet. Specifically, in the liver, the increase of PUFAn3 was evidenced by 3.5-, 2.5-, and 1.5-fold higher levels of EPA (20:5n3), docosapentaenoic acid (DPA, 22:5n3), and DHA, respectively. Concerning the main PUFAn6, there was a significant reduction (−17%) of ARA in a fetus, while no change was observed in the mother. However, a significant increase, in both maternal and fetal liver, of the direct ARA precursor ETA (20:3n6) and a significant reduction of ARA metabolites, as docosatetraenoic acid (DTA, 22:4n6) and DPA (22:5n6), were detected.

**Table 2:** Effect of conjugated linoleic acid-docosahexaenoic acid in phospholipids (CLA-DHA PL) supplementation on fatty acid (FA) profile, expressed in mol%/total FA, in liver and brain of rat dams fed with control (CRTL) and experimental (CLA-DHA PL) diets from day zero to 16 of the gestational period, and their fetuses.

**Fatty acids (FA)**	**Fetal liver**					**Maternal liver**				
	**CRTL**	**SEM**	**CLA-DHA PL**	**SEM**	***P*-value**	**CRTL**	**SEM**	**CLA-DHA PL**	**SEM**	***P*-value**
**ALA, 18:3n3**	0.24	0.02	0.20	0.02	0.13	0.54	0.01	0.44	0.03	**0.02**
SDA, 18:4n3	0.10	0.01	0.11	0.04	0.83	0.13	0.01	0.06	0.01	**0.01**
EPA, 20:5n3	0.21	0.02	0.73	0.07	**0.001**	0.25	0.01	0.84	0.05	**0.0002**
DPA, 22:5n3	0.13	0.01	0.37	0.03	**0.001**	0.54	0.02	0.64	0.04	**0.05**
DHA, 22:6n3	5.86	0.12	9.08	0.40	**0.001**	8.72	0.26	13.16	0.43	**0.0001**
LA, 18:2n6	10.46	0.13	11.47	0.38	**0.05**	18.79	0.46	17.31	0.47	**0.05**
GLA, 18:3n6	0.62	0.01	0.49	0.01	**0.0001**	0.90	0.04	0.46	0.03	**0.00003**
ETA, 20:3n6	1.61	0.02	2.27	0.12	**0.01**	0.34	0.04	0.69	0.07	**0.01**
ARA, 20:4n6	14.46	0.23	11.93	0.25	**0.0001**	16.06	0.79	15.04	0.60	0.34
DTA, 22:4n6	1.92	0.04	1.17	0.05	**0.0001**	0.46	0.02	0.25	0.01	**0.0002**
DPA, 22:5n6	2.22	0.05	0.81	0.04	**0.0001**	0.99	0.09	0.18	0.01	**0.001**
14:1n7	0.41	0.00	0.45	0.08	0.59	0.37	0.02	0.23	0.04	**0.01**
POA, 16:1n7	3.90	0.17	3.70	0.14	0.39	1.57	0.25	1.16	0.11	0.19
OA, 18:1n9	19.97	0.13	18.76	0.14	**0.0005**	15.15	0.96	11.44	0.65	**0.01**
MA, 14:0	1.46	0.02	1.56	0.02	**0.01**	0.37	0.04	0.32	0.02	0.30
PA, 16:0	24.18	0.32	23.78	0.22	0.35	20.37	0.69	20.19	0.55	0.85
SA, 18:0	9.14	0.17	10.12	0.20	**0.01**	14.19	0.75	15.77	0.68	0.16
PUFAn3	6.55	0.16	10.50	0.47	**0.001**	9.61	0.58	15.15	0.41	**0.0001**
PUFAn6	32.50	0.06	28.74	0.41	**0.001**	37.14	0.70	34.14	0.40	**0.01**
MUFA	24.28	0.23	22.92	0.18	**0.003**	17.09	1.20	12.84	0.71	**0.02**
SFA	35.60	0.38	36.50	0.27	0.10	35.97	0.43	37.02	0.31	0.09
PUFAn6/n3	4.97	0.12	2.76	0.15	**0.00001**	3.91	0.18	2.26	0.07	**0.0003**
OA/SA	2.19	0.04	1.86	0.04	**0.001**	1.09	0.13	0.74	0.07	**0.05**
ARA/LA	1.38	0.04	1.05	0.05	**0.001**	0.82	0.04	0.84	0.04	0.79
AI index	0.53	0.01	1.02	0.06	**0.001**	0.58	0.02	0.98	0.03	**0.00001**
	**Fetal brain**					**Maternal brain**				
ALA, 18:3n3	0.19	0.01	0.18	0.01	0.40	0.03	0.00	0.03	0.01	0.45
SDA, 18:4n3	0.20	0.06	0.14	0.03	0.46	ND	ND	ND	ND	ND
EPA, 20:5n3	0.05	0.01	0.10	0.01	**0.02**	0.02	0.00	0.05	0.01	**0.004**
DPA, 22:5n3	0.09	0.02	0.16	0.02	0.07	0.06	0.02	0.07	0.01	0.67
DHA, 22:6n3	4.49	0.05	6.63	0.27	**0.001**	13.32	0.57	14.82	0.40	0.07
LA, 18:2n6	2.25	0.09	2.87	0.10	**0.002**	1.08	0.05	1.18	0.08	0.34
GLA, 18:3n6	0.11	0.01	0.10	0.01	0.52	0.02	0.00	0.02	0.00	0.85
ETA, 20:3n6	0.60	0.07	1.40	0.24	**0.02**	0.49	0.08	0.52	0.04	0.72
ARA, 20:4n6	12.73	0.22	12.45	0.44	0.60	9.39	0.28	9.56	0.17	0.60
DTA, 22:4n6	3.01	0.11	2.73	0.14	0.15	4.25	0.11	4.40	0.12	0.38
DPA, 22:5n6	4.18	0.04	2.73	0.13	**0.0001**	0.82	0.07	0.84	0.09	0.82
14:1n7	0.58	0.14	0.53	0.11	0.78	0.31	0.05	0.41	0.08	0.32
POA, 16:1n7	3.17	0.23	3.25	0.06	0.75	1.14	0.13	1.03	0.12	0.55
OA, 18:1n9	15.78	0.47	16.67	0.42	0.20	20.93	0.68	21.52	1.02	0.65
MA, 14:0	2.49	0.06	2.40	0.05	0.32	1.02	0.20	0.87	0.12	0.59
PA, 16:0	34.61	0.53	32.61	0.32	**0.02**	27.04	0.78	25.11	0.77	0.12
SA, 18:0	11.91	0.15	12.06	0.11	0.44	17.22	0.09	17.08	0.61	0.82
PUFAn3	5.02	0.07	7.16	0.25	**0.001**	13.40	0.57	15.41	0.38	**0.02**
PUFAn6	25.99	0.25	24.22	0.44	**0.01**	37.07	0.96	38.19	1.14	0.47
MUFA	19.53	0.16	20.35	0.47	0.16	1.51	0.08	1.51	0.14	1.00
SFA	48.75	0.05	47.49	0.32	**0.02**	47.98	1.42	45.35	1.21	0.20
PUFAn6/n3	5.18	0.11	3.40	0.15	**0.00003**	2.78	0.08	2.49	0.11	0.07
D9 desat index	1.32	0.04	1.38	0.04	0.35	1.21	0.03	1.28	0.11	0.63
ARA/LA	5.69	0.18	4.35	0.17	**0.001**	0.05	0.01	0.05	0.00	0.84
AI index	0.40	0.01	0.66	0.05	**0.001**	1.47	0.03	1.61	0.02	**0.01**

*Statistical significances (p ≤ 0.05) (in bold) represent the differences of CLA-DHA PL vs. CRTL group (n = 4/group)*.

We observed similar but smaller changes in the PUFA classes in the brain when compared to the liver. As for CLA, we detected a significant increase of EPA in fetuses, while in the PUFAn6 family, there was a significant increment of LA and ETA, but a decrement of DPAn6 metabolite.

Table 2 reports the proportion of the three main FA families, SFA, MUFA, and PUFA (parted in n3 and n6), in the liver and brain of the dams and their fetuses. The total SFA fraction did not change significantly in the liver and brain between dams and their fetuses and between CRTL and CLA-DHA PL fed groups. Focusing on the liver, the CRTL fetuses group displayed higher MUFA and lower total PUFA levels (+40% and −15%, respectively) compared to the dam groups. In the brain, these differences were even more pronounced, as in the fetal group the MUFAs were 9-fold more increased with respect to the dams group, while the total fetal PUFAs were reduced by about 38% when compared to their mothers.

Specifically, as reported in Table 2, the CLA-DHA PL diet significantly decreases hepatic oleic acid (OA, 18:1n9) in the fetus and myristic acid (14:1n7) in the rat dams.

In the liver of the CLA-DHA PL group, the Δ9-desaturation index obtained by the ratio between OA and stearic acid (SA) was significantly decreased by −15% (*p* ≤ 0.001) in fetuses and −32% (*p* ≤ 0.05) in dams, confirming that CLA may regulate Δ9-desaturase enzymatic activity. In addition, CLA-DHA PL supplementation significantly decreased the desaturation index (calculated by the ratio ARA/LA) in fetal liver and brain around −24% (*p* ≤ 0.001), while there was no change in the dams. Another critical parameter is represented by the anti-inflammation index (AI), obtained from (EPA+DHA+ETA)/ARA ratio, which in the CLA-DHA PL rat dams group was significantly incremented of +67% (*p* ≤ 0.0001) and +9% (*p* ≤ 0.01) in liver and brain, respectively and even more in the liver (+91%, *p* ≤ 0.001) and brain (+62%, *p* ≤ 0.001) of their fetuses.

### Hepatic and Cerebral Levels of Different NAEs in Dams and Their Fetuses

Table 3 reports the variations of levels of different NAEs in the liver and brain of the dams and their fetuses induced by maternal intake with CLA-DHA PL diet.

**Table 3:** Effect of conjugated linoleic acid-docosahexaenoic acid in phospholipids (CLA-DHA PL) supplementation on *N*-acylethanolamines (NAEs) profile, expressed in pmol/g tissues, in the liver and brain of rat dams fed with control (CRTL) and experimental (CLA-DHA PL) diets from day zero to 16 of the gestational period, and their fetuses.

***N*-acylethanolamines (NAE)**	**Fetal liver**					**Maternal liver**				
	**CRTL**	**SEM**	**CLA-DHA PL**	**SEM**	***P*-value**	**CRTL**	**SEM**	**CLA-DHA PL**	**SEM**	***P*-value**
POEA	90.35	2.78	69.94	6.59	**0.03**	15.04	0.98	7.39	1.14	**0.001**
AEA	57.76	0.79	41.39	2.52	**0.002**	49.05	7.95	19.91	3.35	**0.02**
DHEA	61.59	3.58	76.61	4.23	**0.03**	33.63	1.46	45.40	3.13	**0.02**
LEA	308.30	9.34	288.43	34.23	0.60	74.00	11.90	34.30	9.52	**0.03**
PEA	296.67	10.24	302.80	35.58	0.88	267.26	18.06	185.43	18.64	**0.01**
OEA	339.44	8.63	279.26	16.13	**0.02**	89.60	2.47	45.37	4.20	**0.0001**
DTEA	19.30	3.35	11.88	1.69	0.11	17.25	1.62	9.12	1.37	**0.01**
SEA	89.68	9.40	79.64	7.18	0.43	131.62	9.89	78.34	6.41	**0.003**
	**Fetal brain**					**Maternal brain**				
POEA	113.69	3.52	109.72	8.32	0.68	8.55	0.79	8.38	0.59	0.87
AEA	27.67	2.46	26.88	2.13	0.82	12.92	0.56	12.93	0.35	0.99
DHEA	34.47	2.75	60.48	3.39	**0.001**	36.66	1.24	34.24	1.60	0.27
LEA	37.15	1.23	42.24	2.05	0.07	3.84	0.24	4.22	0.15	0.22
PEA	186.82	6.84	177.67	8.75	0.44	138.63	10.66	132.05	10.10	0.67
OEA	275.86	6.91	250.56	9.17	0.06	135.93	4.76	138.57	4.85	0.71
DTEA	27.25	1.90	23.44	3.83	0.41	7.74	0.89	8.50	1.10	0.60
SEA	57.45	2.30	66.27	10.01	0.44	25.04	1.54	28.37	2.17	0.25

*Statistical significances (p ≤ 0.05) (in bold) of the differences of CLA-DHA PL vs. CRTL group (n = 4/group)*.

**Figure 4:**
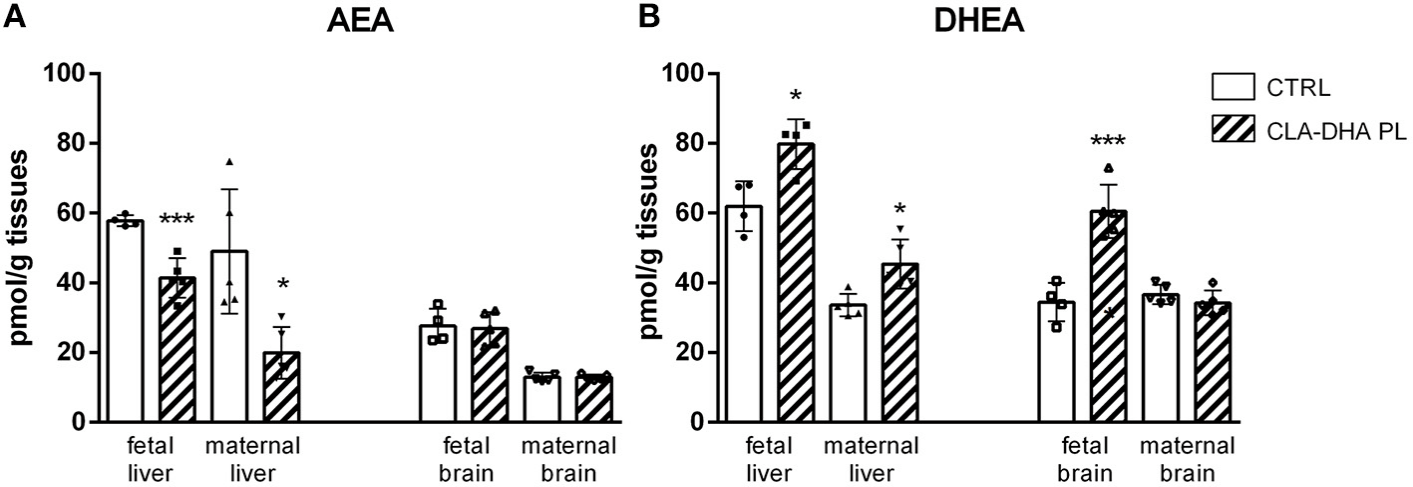
Influence of conjugated linoleic acid-docosahexaenoic acid in phospholipids (CLA-DHA PL) diet on modification of *N*-arachidonoylethanolamine (AEA) **(A)** and *N*-docosahexaenoylethanolamine (DHEA) **(B)** levels, expressed in pmol/g tissues, in the liver and brain of rat dams fed with control (CRTL) or experimental diets (CLA-DHA PL) from day 0 to 16 of the gestational period, and their fetuses (*n* = 4/group). Statistical significance is depicted as follows: *p ≤ 0.05 and ***p ≤ 0.001.

In particular, in fetal and maternal liver, CLA-DHA PL diet significantly decreased concentrations of *AEA*, the ARA-derived NAE, when compared to the control group (Figure 4A), while there was no change in the brain. On the contrary, in both fetal and maternal liver and fetal brain of CLA-DHA PL groups, there was a significant increase of the DHA-derived NAE (Figure 4B).

## Discussion

Brain development is critically dependent on FA availability during fetal life (58). Thereby maternal nutritional status should be adequate to ensure a proper fetal FA supply *via* transplacental transport (59, 60). One of the emerging properties of DHA on neurodevelopment may be linked to its metabolite DHEA, which has been shown to promote synaptogenesis and neuritogenesis (61). Therefore, it appears essential to correctly balance the amounts and relative proportions of PUFAn3 and n6 in the mother's diet during the perinatal period to modulate the lipidic status efficiently and subsequently the metabolic pathways in various tissues of the progeny (62, 63).

In this study, we utilized dietary PL-bound CLA and DHA in rat dams during the first 2/3 of their pregnancy at a concentration that can be translated, in humans, to 3g/d CLA and 800 mg of DHA attainable by dietary supplements (48).

Our data showed that CLA and DHA in PL form crossed the placenta and were readily incorporated into the maternal and fetal liver and brain of fetuses, but not into dams' brain, accordingly to previous studies where short-term dietary supplementation with CLA or DHA was hardly incorporated into adult rat brain (56, 58). Furthermore, we surprisingly observed that CLA metabolites, the CD16:2 and CD18:3, were also incorporated into these fetal tissues. Interestingly, we observed a significant positive correlation between circulating levels of maternal CLA and its CD18:3 metabolite with the respective levels in fetal liver (R = 0.72; *p* ≤ 0.03 and 0.83 *p* ≤ 0.01, respectively), while we did not find any significant correlation with CD16:2 metabolite, which is produced by CLA peroxisomal beta-oxidation. These data indicate that CLA is probably desaturated to CD18:3 in the maternal but not in fetal liver and crosses the placenta successively, while the peroxisomal beta-oxidation might occur in the fetus and the CLA-DHA PL diet might over-activate this process. We observed that CLA and DHA intake, by reducing PUFAn6 and increasing PUFAn3, led to a significant reduction of PUFAn6/n3 ratio in fetal liver (−44%) and brain (−34%) and in the dams' liver (−42%). The reduction in FA belonging to the n6 family observed in the CLA-DHA PL groups could be due not only to dietary DHA but CLA may also contribute either by competing with LA for the desaturase and elongase enzymes, as shown in different studies (64) or by enhancing DHA biosynthesis (29).

Further evidence of possible competition between CLA and LA in fetal liver and brain is suggested by a significant decrease of the desaturation index, calculated by ARA/LA ratio. Moreover, the DPAn6 reduction might derive from competition in peroxisomes for beta-oxidation between CLA and 24:5n6, the direct precursor of DPAn6, as CLA was promptly and abundantly beta-oxidized. As expected, the lack of incorporation of DHA, CLA, and its metabolites into the maternal brain resulted in no changes in FA families, in opposition to fetal brain.

In addition, we reported that the CLA-DHA PL intake might be protective vs. inflammation by modulating the n6/n3 ratio, leading to an increase of the AI in the liver and brain of dams and their fetuses. Indeed, CLA has been shown to possess neuro-anti-inflammatory properties by activating PPAR alpha (34). It is possible to speculate about a fetal capacity to react during an intra-uterine inflammation, predisposing the fetus to hypoxic stress and thereby brain damage (65).

CLA-DHA PL intake also caused a significant decrease of hepatic MUFA, in particular OA level in the fetus, probably due to a downregulation of Δ9 desaturase (stearoyl-coenzyme A-desaturase, SCD) activity, as confirmed by a reduction of OA/SA ratio (66). In a previous study on obese Zucker rats fed with CLA, we observed a reduced hepatic Δ9 desaturase index strongly correlated to PUFAn3 (50); the expression of the SCD gene was also downregulated by a lower PUFAn6/n3 ratio (67–71). Physiologically, a downregulated SCD activity may prevent triglyceride accumulation in the liver (70) particularly in the fetus, where an enhanced *de novo* lipogenesis may favor steatosis.

*N*-acylethanolamines are bioactive lipid mediators, whose biosynthesis is influenced by dietary FA composition, belonging to the endocannabinoidome (eCBome), a system widely distributed in various tissues and organs able to modulate numerous physiological functions, neuroprotection, and inflammation (28, 34, 46–50). Furthermore, the eCBome is involved in the regulation of fetal neurogenesis and it has been demonstrated that an altered cannabinoid signaling can exert long-lasting consequences in neuronal functions of the adult brain by modifying neurodevelopment (72, 73). Our data indicate that the CLA-DHA PL diet significantly decreased AEA levels in fetal and maternal liver and increased DHEA both in the fetal and maternal liver and the fetal brain. The biosynthetic pathway for the production of AEA and DHEA might be influenced by the reduced tissue ARA/DHA ratio as previously demonstrated (47). Noteworthy, DHEA is a key active metabolite of DHA, and it has been shown that its content in the fetal hippocampus decreased following the reduction of DHA through maternal dietary depletion of PUFAn3 (74); on the contrary, DHEA levels can be increased by dietary inclusion of DHA (49). It has been shown that DHEA promotes neurite growth, synaptogenesis, and expression of glutamate receptor subunits, thus enhancing glutamatergic synaptic activity, which stimulates the development of hippocampal neurons (74). The formulation used in our study, where CLA was simultaneously esterified in PL with DHA, may be advantageous because the association of CLA and DHA, readily incorporated in the fetus CNS, might exert anti-inflammatory function via PPAR alpha activation and enhanced neurogenesis. Therefore, the administration of CLA and DHA could be considered a dietary treatment during pregnancy able to protect the offspring from neurological and psychiatric disorders with neuroinflammatory and neurodegenerative basis during the critical prenatal period.

## Data Availability Statement

The raw data supporting the conclusions of this article will be made available by the authors, without undue reservation.

## Ethics Statement

The animal study was reviewed and approved by the Animal Ethics Committees of the University of Cagliari and were carried out in accordance with the European Directive on the protection of animals used for scientific purposes (2010/63/EU).

## Author Contributions

EM, GC, MP, and SB contributed to conception and design of the study. EM, GC, and CM performed the lipid analysis. MS and RM performed the animal treatment. EM, GC, CM, MP, RM, and SB performed data analysis and wrote sections of the manuscript. All authors contributed to manuscript revision, read, and approved the submitted version.

## Funding

This work was supported by a grant, with the code POC01_00069 from the Italian Ministry of University and Research (MUR) within the program Proof of Concept.

## Conflict of Interest

AS was employed by Innolipid AS. As a potential conflict of interest SB and MP are co-inventors of the patent WO2016/016790 A1, of the University of Cagliari, by the title: Ester of a phospholipid with conjugated linoleic acid for the treatment of psychiatric disorders with neuroinflammatory and neurodegenerative basis. The remaining authors declare that the research was conducted in the absence of any commercial or financial relationships that could be construed as a potential conflict of interest.

## Publisher's Note

All claims expressed in this article are solely those of the authors and do not necessarily represent those of their affiliated organizations, or those of the publisher, the editors and the reviewers. Any product that may be evaluated in this article, or claim that may be made by its manufacturer, is not guaranteed or endorsed by the publisher.
